# Portable Fire Escape

**Published:** 1895-03-30

**Authors:** 


### PRACTICAL DEPARTMENTS.

### PORTABLE FIRE ESCAPE.

In spite of the many ingenious, and, on the whole, inexpensive, contrivances which exist for facilitating escape from upper storeys in case of fire, it does not seem that they are as widely in request as might be wished, and from the accounts of most of the terrible disasters from fire which are constantly appearing in the newspapers, it is usually apparent that even the simplest precaution, such as the provision of at least a strong piece of rope, is usually neglected by the ordinary householder. It is quite certain that every house should be provided with something in the nature of a fire escape, more particularly when the nearest fire station is some distance off, as in country places. The only difficulty is to decide on the best apparatus. That perhaps best known is the "Chute" kind, which is simple and good, but some people prefer a ladder arrangement, and we have lately seen one of this description which would appear to answer every purpose. It is patented by Mr. J. W. Palmer, and may be obtained from Mr. W. Love, of Union Street, Borough. It consists of a chain ladder, which when not in use can be stowed away into a very moderate sized box, just inside the window. There are steps inside the lid of the box by which to climb on to the sill. When the ladder is to be used, it is payed out of the window, being carried easily down by its own weight. At the ends of the chain ladder ropes are attached, enabling the whole apparatus to be fixed from below at any angle from the window. The ladder gives a firm and safe means of descent, and will, we think, be found a very satisfactory arrangement, occupying not much space, and forming a prompt and efficient exit in case of fire. The price complete is from 3s. per foot.

